# The Pathophysiological Mechanisms and Pattern of Dyslipidemia Associated with Iodine Deficiency and Subclinical Hypothyroidism in Pregnant Normotensive and Preeclamptic Central African Women

**DOI:** 10.3390/pathophysiology32020018

**Published:** 2025-04-18

**Authors:** Charles Bitamazire Businge, Benjamin Longo-Mbenza

**Affiliations:** 1 Department of Obstetrics and Gynaecology, Faculty of Health Sciences, Walter Sisulu University, Private Bag x1 WSU, Mthatha 5117, South Africa; 2 Faculty of Medicine, University of Kinshasa, Kinshasa, Democratic Republic of the Congo; longombenza@gmail.com; 3 Department of Public Health, Lomo University of Research, 652 Freesias, Kinshasa, Democratic Republic of the Congo

**Keywords:** dyslipidemia, iodine deficiency, sub-clinical hypothyroidism, normotensive pregnancy, preeclampsia

## Abstract

Background: Pregnancy simulates a metabolic syndrome-like state and predisposes to iodine deficiency and hypothyroidism through increased iodine renal loss and transplacental transfer to the fetus. Iodine deficiency is thought to predispose to dyslipidemia through elevation of serum TSH. Obesity, dyslipidemia, and hypothyroidism are established risk factors of preeclampsia. Hence, pregnant women with iodine deficiency are likely to be at increased risk of dyslipidemia and preeclampsia. We investigated the pattern of dyslipidemia among preeclamptic and normotensive pregnant women with and without iodine deficiency. Methods: The pathophysiological mechanisms linking iodine deficiency and dyslipidemia were delineated using bivariate correlations, logistic regression, and exploratory factor analysis of anthropometric, lipid profile, urine iodine concentration (UIC), and thyroid function data from 240 women with preeclampsia and 120 normotensive pregnant controls at term who attended Lomo Medical Centre, Democratic Republic of Congo (DRC). Results: Preeclamptic women with iodine deficiency had significantly lower HDL-C but higher triglyceride levels than those with sufficient iodine intake. Both normotensive and preeclamptic participants with elevated TSH had high serum oxidized LDL-C but low NO, *p* < 0.001. Conclusions: SCH, secondary to iodine deficiency, is associated with elevated serum oxidized LDL and decreased Nitric Oxide (NO) among both normotensive and preeclamptic women, while insufficient iodine nutrition among preeclamptic women predisposes to reduced HDL-C and increased serum Triglycerides, which are risk factors of atherosclerosis and cardiovascular disease.

## 1. Introduction

The physiological changes in lipid metabolism during pregnancy are characterized by estrogen and progesterone-mediated enhancement of lipid synthesis and storage in the first half of pregnancy for use as the preferred substrates for maternal metabolism in the second half of pregnancy [1,2]. Hence, triglycerides increase by 200–400% in a normal pregnancy, LCL-C and HDL-C by about 25–50% [3,4]. This increase, observable after the 6th week of gestation, reaching a peak in the second trimester, ensures the accumulation of adequate maternal lipid substrates for fetal growth and development and maternal energy requirements in the third trimester [4,5].

This physiological increase in lipid synthesis during pregnancy is coupled with a decrease in the size of LDL-C particles with resultant high concentrations of small-dense LDL-C (sdLDL-C), a precursor of atherogenesis [5,6]. In normal pregnancy, the atherogenic risk arising from high plasma sdLDL-C is alleviated by increased apolipoprotein A-I (Apo A-I) and HDL-C that attain maximum levels in the second trimester [5].

In the second half of pregnancy, there is a transition from active lipid synthesis in the liver to more significant catabolism and utilization of stored fats as fuel for maternal metabolic processes instead of glucose [7,8]. This transition is mediated by a physiological increase in insulin resistance that facilitates a regular supply of glucose from the maternal circulation to the fetus. This enhanced lipolysis of stored fats increases the serum levels of free fatty acids and glycerol. This is also attributed to the simultaneous reduction in lipoprotein lipase activity that prevents the reintegration of the triglycerides into the adipose tissue.

Only limited amounts of free fatty acids and glycerol are transferred to the fetus by the placenta; hence the greater bulk is re-esterified in the liver and packaged into very low-density lipoprotein cholesterol (VLDL-C), accounting for the significantly increased levels of maternal serum total cholesterol, Triglycerides, LDL-C as well as Lipoprotein(a) [Lp(a)] in the latter half of pregnancy [5,8]. Women with serum triglyceride levels above 1.7 mmol/L, even in the absence of concurrent cardiovascular disease, are at increased risk of incident preeclampsia and gestational diabetes [9]. This was corroborated in a large cohort study that reported elevated preconception serum levels of triglycerides, LDL-C, and diminished serum HDL-C levels as risk factors for incident preeclampsia [10]. This implies that hypertriglyceridemia heightens the degree of endothelial dysfunction and insulin resistance, which are the main pathological mechanisms for preeclampsia and gestational diabetes, respectively.

For a long time, the elevation of serum lipids was thought to be physiological, with no associated maternal or fetal adverse outcomes. It is now recognized that extreme elevation of total cholesterol, LDL-C, and triglycerides, as well as markedly low HDL-C, predispose to adverse maternal outcomes such as abruptio placenta, preeclampsia, preterm delivery, and higher risk for future atherogenic cardiovascular diseases for both mothers and babies [8,11,12]. Other fetal complications, such as intrauterine growth restriction and fetal distress, may arise secondary to maternal systemic endothelial dysfunction that also diminishes placental function [5].

Excessive increase in serum lipids during pregnancy is more likely among women who are already at risk of dyslipidemia, such as familial dyslipidemia syndrome, and others with conditions such as DM and hypothyroidism [13]. Iodine deficiency is the primary cause of overt and subclinical hypothyroidism globally. Several studies have reported that inadequate iodine nutrition status is associated with hypertriglyceridemia, hypercholesterolemia, preeclampsia, and hypertension [14,15]. Iodine supplementation and a diet rich in iodine are associated with a reduced risk of dyslipidemia [16,17]. However, other studies reported a U-shaped relationship between iodine nutrition status and dyslipidemia [18,19]. Both inadequate and excessive iodine intake can predispose to hypothyroidism, characterized by elevated serum TSH.

Pregnancy predisposes to insufficient iodine nutrition through increased physiological demand and renal filtration [20,21]. Iodine deficiency is also thought to predispose to dyslipidemia, with the proposed mechanism being the elevation of serum TSH as it occurs in overt and subclinical hypothyroidism [22]. Pregnancy simulates a metabolic syndrome-like state and is associated with increased levels of serum lipids, especially in the third trimester and among obese women [8]. Hence, pregnant women with obesity and iodine deficiency are likely to have abnormal lipid profiles. Obesity, dyslipidemia, and hypothyroidism are known risk factors of preeclampsia [23,24].

Pre-eclampsia is a complex and life-threatening multisystem disorder characterized by sudden-onset hypertension after 20 weeks of gestation and at least one other associated complication, including proteinuria, maternal organ dysfunction, or uteroplacental dysfunction [25]. The etiology of preeclampsia is broadly categorized into two. The first (the placental etiology) is characterized by abnormal trophoblastic invasion of the myometrial spiral arteries, placenta ischemia, and inappropriate over-secretion of soluble fms-like tyrosine kinase-1 (sFlt-1). sFlt-1 is a soluble receptor for the vascular endothelial growth factor (VEGF) that binds to Placental Growth factor (PlGF) and VEGF receptors, thereby blocking their angiogenic function in the maternal circulation, leading to early onset preeclampsia before 34 weeks of gestation [26]. The second is the interaction between a healthy placenta and pre-pregnancy maternal factors predisposing to endothelial dysfunction and causing microvascular damage. These include nutritional deficiencies such as iodine, vitamins A, C, and E, and chronic maternal illnesses such as diabetes mellitus, systemic lupus, antiphospholipid syndrome, chronic hypertension, renal disease, and obesity [27]. The unifying pathological pathway between these two etiologies is oxidative stress that leads to lipid peroxidation and damage to DNA and protein moieties, which the use of a balanced diet can alleviate, and among the populations with diets deficient in micronutrients and vitamins, the use of nutraceuticals [28,29].

It is not certain whether iodine deficiency is an independent predictor of dyslipidemia. The pathological mechanisms through which iodine deficiency may independently predispose to dyslipidemia have not yet been described. This study set out to establish whether insufficient iodine intake is an independent risk factor for dyslipidemia among preeclamptic and normotensive pregnant women with or without iodine deficiency and/or subclinical hypothyroidism and the likely pathophysiological mechanisms involved.

## 2. Materials and Methods

### 2.1. Study Setting

Lomo Medical Centre (LMC) is a tertiary hospital in Kinshasa province of the Democratic Republic of Congo. The Democratic Republic of Congo has the world’s third highest age-standardized rate of iodine deficiency, estimated at 16,385/100,000 people [30].

### 2.2. Study Design

This analytical cross-sectional study was carried out as a secondary analysis of archived data from a cohort of women enrolled in the Communicable Disease, Nutritional, Environmental Epidemiological Risk study (CDNER) carried out in Kinshasa Province, Democratic Republic of Congo, and coordinated at Lomo Medical Centre between 2007 and 2008. The study was approved by the Lomo Medical Centre Institutional Review Board (Reference no. LMDE031LMB02).

The sociodemographic characteristics and lipid profiles of 120 normotensive pregnant women randomly enrolled in the primary study by systematic random selection and 240 cases of preeclampsia who were consecutively enrolled were used for the current study. The normotensive controls were pregnant women admitted at term for elective cesarean section and counterparts in latent labor at term admitted for vaginal delivery. They were enrolled at term just before delivery in order to avoid potential misclassification of women capable of developing late-onset preeclampsia as controls. The cases were women with preeclampsia diagnosed according to the International Society for the Study of Hypertension in Pregnancy (ISSHP) guidelines. Preeclampsia was defined as the new onset of hypertension after 20 weeks of gestation with SBP > 140 mmHg systolic or DBP > 90 mmHg diastolic, measured at least 4 h apart, or a single SBP > 160 mmHg or DBP > 110 mmHg with proteinuria or other features of end-organ damage [31].

The participants’ height, weight, and systolic and diastolic blood pressure were measured according to standardized procedures. Overnight fasting venous blood was drawn from the cubital fossa between 7:00 and 9:00 a.m. The blood samples were assayed immediately to measure the concentrations of high-density cholesterol (HDL), total cholesterol, triglycerides, low-density lipoprotein (LDL), and oxidized low-density lipoprotein (oxLDL). Laboratory data were obtained using calibrated and standard routine procedures and specific protocols of manufacturers, such as kits of Biomérieux (Marcy l’Etoile, France) and Mercodia AB (Silveniusgatan 8 A, SE754, Uppsala, Sweden) and a caloric Sensor Hach DR/2010 spectrophotometer (HACH, Loveland, CO, USA). T3, T4, and TSH were measured by the enzyme-linked immunosorbent assay method purchased from DIALAB GmbH IZ-NOE Sued Company, Hondastrasse, Objekt M55, A-2351 wr, Neudorf, Austria. Urinary iodine concentration was measured using the Sandell–Kolthof method.

### 2.3. Statistical Analysis

Data analysis was performed using the software package IBM SPSS STATISTICS version 29 for Windows (IBM Inc., Chicago, IL, USA). The data were summarized into proportions (%) for categorical variables, means ± standard deviation (SD) for normally distributed variables, and median (25th–75th percentiles) for non-normally distributed continuous variables, respectively. The Chi-square test was used to compare categorical variables between groups. The student’s *t*-test was used to compare means between two groups, and one-way analysis of variance (ANOVA) was used to compare means involving more than two groups. Univariate Odds ratios (OR) were computed for the association between potential contributing factors and preeclampsia. A *p* < 0.05 was considered significant. An exploratory component analysis was conducted to delineate the patterns of interaction between UIC, TSH, HDL-C, LDL-C, oxidized LDL-C, and nitric oxide in participants with preeclampsia. Eigenvalues ≥ 1 were used to identify the main latent interactions of these variables among participants with preeclampsia in the study population.

## 3. Results

### 3.1. General Characteristics of the Participants

The mean chronological ages were 32.4 ± 6.0 and 34.0 ± 4.5 years, and the mean gestational ages were 30.8 ± 7.9 and 38.6 ± 2.3 weeks’ gestation, respectively, for preeclamptic and normotensive women. Eighty-seven percent (209/240) of the preeclamptic and 27% (32/120) of the normotensive pregnant women had insufficient iodine nutrition (UIC < 150 µg/L) (*p* < 0.0001). Seventy-five percent (180/240) of the preeclamptic women and 54.2% (65/120) of the normotensive women had TSH above the upper range of normal third-trimester pregnancy levels of 3.0 UI/L (*p* < 0.0001).

Normotensive pregnant women had higher HDL-C, LDL-C, and nitric oxide, while preeclamptic participants had higher BMI, oxidized LDL-C, and triglycerides (Table 1).

### 3.2. The Relationship Between Iodine Nutrition Status, TSH, Nitric Oxide, and Serum Lipids

Both preeclamptic and normotensive pregnant women with elevated TSH had higher oxidized LDL-C and lower nitric oxide levels than respondents with TSH levels within normal pregnancy levels. HDL-C was significantly lower, while LDL-C and triglycerides were significantly higher among preeclamptic women with insufficient iodine nutrition status (UIC < 150 µg/L) than those with sufficient iodine nutrition status (Table 2 and Table 3).

UIC positively and strongly correlated with HDL-C and showed a moderate positive correlation with LDL-C, Triglycerides, and NO, as well as a mild to moderate negative correlation with TSH and oxidized LDL. TSH was positively correlated with oxidized LDL-C but negatively correlated with NO, HDL-C, LDL-C, and UIC. A strong and negative correlation existed between NO (an antioxidant biomarker) and TSH and oxidized LDL-C (Table 4).

Univariable and multivariable logistic regression were conducted to establish the independent predictors of low HDL (below the lower range of normal, 40.3 mg/dL). Preeclampsia status and TSH > 3 IU/L were independent predictors of low serum HDL. UIC >150 µg/L. was protective against low HDL (Table 5).

**Figure 1:**
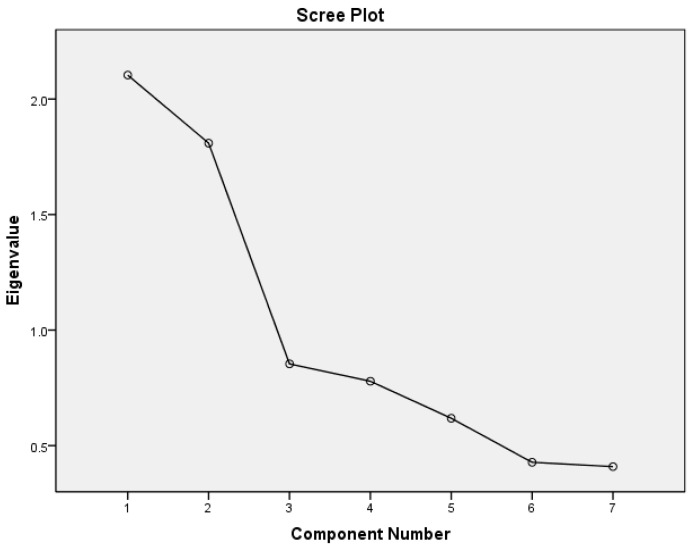
A scree plot for participants with preeclampsia showing components with corresponding Eigenvalues.

Exploratory factor analysis revealed two significant patterns through which UIC, thyroid function parameters, and lipid profile variables interacted in participants with preeclampsia. The first includes the urine iodine concentration that correlates positively with HDL cholesterol but negatively with triglycerides and LDL, and the second involves a positive correlation between serum TSH and oxidized LDL but low serum NO (Figure 1 shows components 1 and 2 in the scree plot, as well as in Table 6 below).

## 4. Discussion

This study finds that preeclampsia was associated with low UIC, elevated TSH, and higher but normal levels of T3 and T4, which are features consistent with chronic iodine deficiency, thyroid hyperstimulation, and subclinical hypothyroidism. The serum levels of HDL-C and the UIC of preeclamptic women were much lower than those of normotensive women. Furthermore, insufficient iodine intake in pregnancy was associated with low HDL-C and elevated triglycerides only among preeclamptic women. Exploratory factor analysis for patterns of association revealed that a sufficient iodine nutrition status is associated with higher serum levels of HDL-C and lower levels of LDL-C and triglycerides (latent factor 1 in Figure 1). These findings suggest that iodine may be an essential factor in HDL-C’s synthesis or metabolism. A sufficient iodine nutritional status in pregnancy is associated with a less atherogenic lipid profile and a normotensive state.

The elevated serum LDL-C and triglycerides in the current study may have resulted from low serum HDL-C, which was associated with an insufficient iodine nutrition status. HDL cholesterol is known for transporting LDL-C and VLDL cholesterol from the circulation towards the liver. High serum concentrations of LDL-C and VLDL cholesterol result in lipid peroxidation and endothelial dysfunction [32], increasing the risk of preeclampsia. According to the World Health Organization criteria, the median UIC of 98 µg/L of the preeclamptic participants in the current study reveals an insufficient iodine nutrition status [33]. Furthermore, Iodine is one of the exogenous antioxidants; hence, the level of iodine deficiency among preeclamptic participants in the current study may partially explain the concurrent elevation in serum oxidized LDL-C observed in the current study [34].

The findings of the current study are consistent with those of previous studies. In two clinical trials and observation studies [16,35,36,37], iodine supplementation could only reduce the level of LDL-C without a significant impact on HDL-C or triglycerides [16,35]. However, in both clinical trials, all the participants’ UIC remained well below 100 µg/L, the cut-off value for adequate iodine nutrition [38] after the iodine supplementation period. The findings in the current study may imply that only individuals with sufficient iodine intake during pregnancy will have HDL-C within the normal range.

Previously, it has been suggested that the mechanism by which iodine deficiency predisposes to low HDL-C is through elevated TSH [39,40]. However, the results of the current study suggest that iodine deficiency may increase the risk of low serum HDL-C independent of TSH. Although this phenomenon in the current study was more pronounced among participants with preeclampsia, the results are concordant with findings reported by Kim et al. [19] and Wang et al. [36], who, respectively, reported that a diet supplemented with iodine-rich seaweeds and higher water iodine content was associated with higher serum HDL-C levels [19,41]. This phenomenon may be explained by reduced serum antioxidant capacity among people with low iodine nutrition status, which reduces serum anti-inflammatory capacity and increases the risk of oxidative stress [34,42]. Systemic inflammation is associated with reduced HDL-C synthesis. At the same time, oxidative stress depletes serum HDL-C levels by scavenging and delivering lipid peroxides from VLDL-C to the liver, thus preventing the deposition of atherogenic lipids in the vessels [43,44]. During normal pregnancy, there is a progressive increase in VLDL-C, LDL-C, and triglyceride serum levels from the first to the third trimester. In contrast, HDL cholesterol increases from the first to the second but tends to decline slowly in the third trimester, but it does not reach pre-pregnancy levels [45,46]. In addition, there is a progressive rise in oxidized lipids, but this is variably matched with antioxidant enzymatic activity [45,46].

Both normotensive and preeclamptic women had median TSH above the upper range expected in the third trimester despite T3 and T4 in the normal range, which is consistent with subclinical hypothyroidism [47]. Although only the preeclamptic women had median UIC consistent with insufficient iodine nutrition status, the median UIC of the normotensive pregnant women was near the lower limit of the normal range. This implies a high prevalence of subclinical hypothyroidism in the study population secondary to insufficient iodine nutrition in pregnancy, especially among preeclamptic women.

In the current study, high serum TSH levels were associated with high serum oxidized LDL cholesterol and low nitric oxide levels among preeclamptic and normotensive women. Chronic iodine deficiency leads to persistent thyroid gland TSH stimulation, predisposing to increased production of hydrogen peroxide and superoxide radicals [48,49]. Hydrogen peroxide and superoxide radicals react with and rapidly diminish Nitric Oxide (NO) levels, forming peroxynitrite (ONOO-). This more potent oxidant can cause lipid oxidation and accentuate endothelial dysfunction [50]. Furthermore, elevated serum TSH stimulates extra-thyroidal endothelial-TSH receptors, leading to increased tumor necrosis factor alpha (TNF-α) and endothelin production and reduced prostacyclin and nitric oxide production [51]. Low serum NO predisposes to endothelial dysfunction, reduction in flow-mediated dilatation, endothelial activation, and increased carotid media-intima thickness (cIMT), pathological features of preeclampsia [52,53,54,55,56,57,58] and atherosclerosis, which is a herald of cardiovascular disease [59,60]. These mechanisms may account for the high levels of oxidized LDL-C and low levels of Nitric Oxide observed among participants with preeclampsia in the current study.

The association between insufficient iodine nutrition, hypothyroidism, and preeclampsia observed in the current study implies that ensuring adequate iodine intake among women in reproductive years may reduce the incidence and severity of preeclampsia and associated complications such as acute kidney injury, postpartum hemorrhage, chronic hypertension, and future cardiovascular disease [61,62].

Given the risk of dyslipidemia arising from iodine deficiency and resultant gestational and long-term consequences such as cardiovascular disease, it is prudent that women at risk of iodine deficiency in pregnancy receive iodine supplementation [63,64]. However, to avoid adverse effects associated with potential excessive iodine intake following iodine supplementation, such as thyroid nodules, autoimmunity, and iodine-induced hypothyroidism, it is recommended by the World Health Organization that iodine supplementation during pregnancy should not be implemented when the iodine nutrition status of the general has been sufficient (median UIC ≥ 100 μg/L) for a period of ≥2 years [65]. Women in reproductive years at risk of dyslipidemia secondary to thyroid autoimmunity refractory to conventional treatment may benefit from stem-cell therapy that has shown some promise. This will be informed by ongoing research in this field [66,67].

Other potential remedies for women at risk of severe dyslipidemia in pregnancy that could be used alone or in combination with iodine supplements include alpha lipoic and inositol isomers. Alpha lipoic acid has been reported to reduce the serum levels of total cholesterol, triglycerides, and LDC-C but had no significant effect on HDL-C [68,69,70]. These could help mitigate the adverse effects of excessive iodine intake.

Since the increased synthesis of triglycerides and LDL-C in pregnancy is mediated through elevation of insulin resistance in the second half of pregnancy, myoinositol and D-chiro inositol, the two inositol isomers that have shown effectiveness in increasing insulin sensitivity and restoring ovulation in women with the polycystic ovarian syndrome and reduction of follicle-stimulating hormone among menopausal women may have a future role in the management of women at risk of hyperlipidemia in pregnancy [71,72].

Furthermore, myoinositol may confer other health benefits, such as reducing breast density, which, when elevated, increases the risk of breast cancer [73]. This effect is enhanced when myoinositol is administered with other nutraceuticals, such as boswellic acid and betaine. The proposed mechanisms of action are the synergistic anti-inflammatory and endocrine modulation [73,74], which likely mitigates the lipid peroxidation and reduces total antioxidant capacity observed among women with breast carcinoma [75]. Hence, it is recommended that health professionals take an appropriate dietary history to recognize and correct micronutrient deficiencies with a balanced diet as a long-term measure and nutraceuticals where applicable [76].

## 5. Conclusions

Insufficient iodine nutrition in pregnancy is associated with subclinical hypothyroidism and preeclampsia. Among both normotensive and preeclamptic women, subclinical hypothyroidism predisposes to elevated serum oxidized LDL-C and decreased NO. In contrast, insufficient iodine nutrition among preeclamptic women predisposes them to reduced HDL-C and increased serum Triglycerides, which are risk factors for atherosclerosis and cardiovascular disease. Iodine supplementation for women at risk of iodine deficiency in pregnancy, especially those with risk factors for preeclampsia and dyslipidemia, may help avert complications during pregnancy and future cardiovascular disease. Correcting other nutritional deficiencies using a balanced diet and nutraceuticals when feasible may further improve the general health of these women, especially the prevention of oxidative stress-mediated chronic conditions such as hypertension, diabetes mellitus, and cancers.

## Figures and Tables

**Table 1:** The mean and medians of various biomarkers of iodine nutrition status, thyroid function, and lipid profile of the participants.

	Preeclamptic	Normotensive	
Variable	Mean ± SD or Median (IQR)	Mean ± SD or Median (IQR)	*p*
UIC µg/L	109.2 ± 31.1	161.8 ± 24.1	<0.0001
TSH mIU/L	5.3 ± 2.6	4.0 ± 3.2	<0.0001
T3 ng/mL	1.46 ± 0.40	1.15 ± 0.28	<0.0001
T4 µg/mL	11.21 ± 2.56	9.70 ± 2.32	<0.0001
BMI Kg/m^2^	25.3 ± 6.2	22.2 ± 5.4	<0.0001
HDL-C mg/dL	21.3 ± 12.7	52.6 ± 2.9	<0.0001
LDL-C mg/dL	45.9 ± 11.0	56.5 ± 3.1	<0.0001
oxLDL-C UI/L	189.0 (98.0, 222.0)	87.5 (21.2, 189.0)	<0.0001
Trigl mg/dL	30.75 (20.00, 102.72)	19.83(19.82, 19.83)	<0.0001
NO µmol/L	3.0 (2.0, 7.0)	7.0 (1.0, 32.0)	<0.0001

Trigl, triglycerides; oxLDL, oxidized low-density lipoprotein.

**Table 2:** Comparison of serum levels of HDL-C, LDL-C, Triglycerides, oxidized LDL-C, and nitric oxide of preeclamptic and normotensive participants stratified according to serum TSH > or ≤ 3 mIU/mL.

	Preeclamptic Participants			Normotensive Participants		
	TSH ≤ 3 mIU/L n = 60	TSH > 3 mIU/L n = 180		TSH ≤ 3 mIU/L n = 55	TSH > 3 mIU/L n = 65	
Variable	Mean ± SD or Median (IQR)	Mean ± SD or Median (IQR)	*p*-Value	Mean ± SD or Median (IQR)	Mean ± SD or Median (IQR)	*p*-Value
HDL-C mg/dL	20.5 (10.0, 30.9)	20.0 (10.0, 28.6)	0.763	50.3 ± 2.0	52.2 ± 3.7	0.313
LDL-C mg/dL	46.0 ± 11.3	45.8 ± 11.0	0.934	56.9 ± 2.1	56.2 ± 3.9	0.189
oxLDL-C UI/L	93.5 (19.9, 222.8)	190.0 (110.3, 222.0)	<0.001	29.9 (9.0, 58.0)	108.4 (46.9, 211.0)	<0.001
Trigl mg/dL	52.1 ± 11.3	52 ± 11.0	0.933	63.0 ± 2.0	63.0 ± 4.0	0.213
NO µmol/L	7.0 (4.0, 27.0)	2.0 (1.0, 6.0)	<0.001	32.0 (20.3, 44.0)	2.0 (1.0, 3.0)	<0.001

**Table 3:** Comparison of serum levels of HDL, LDL, Triglycerides, oxidized LDL, and nitric oxide of preeclamptic and normotensive participants stratified according to UIC levels ≥ or < UIC 150 µg/L.

	Preeclamptic Participants			Normotensive Participants		
	UIC > 150 µg/L n = 31	UIC < 150 µg/L n = 209		UIC > 150 µg/L n = 88	UIC < 150 µg/L n = 32	
Variable	Mean ± SD or Median (IQR)	Mean ± SD or Median (IQR)	*p*-Value	Mean ± SD or Median (IQR)	Mean ± SD or Median (IQR)	*p*-Value
HDL-C mg/dL	35.0 (28.0, 45.0)	14.0 (10.0, 28.0)	<0.001	52.9 ± 1.9	51.6 ± 4.6	0.036
LDL-C mg/dL	42.0 ± 8.0	47.0 ± 11.0	0.027	56.9 ± 2.1	55.3 ± 4.9	0.036
oxLDL-C UI/L	191.2 (89.0, 234.0)	189.0 (95.6, 222.0)	0.436	87.5 (21.2, 189.0)	87.5 (18.7, 186.3)	0.808
Trigl mg/dL	48.0 ± 10.0	53.0 ± 11.0	0.027	63.2 ± 2.1	61.8 ± 5.0	0.360
NO µmol/L	3.0 (1.0, 5.0)	3.0 (2.0, 7.5)	0.472	3.5 (1.0, 32)	16 (2.0, 39)	0.132

**Table 4:** Bivariate correlation between NO, TSH, HDL, LDL, and Oxidized LDL.

	Pearson	UIC	HDL	LDL	Trigl	TSH	NO	oxLDL-C
UIC	correlation		0.729 **	0.239 **	0.239 **	−0.133 *	0.207 **	−0.215 **
	*p*-value		<0.001	<0.001	<0.001	0.011	<0.001	<0.001
HDL-C	correlation	0.729 **		0.214 **	0.214 **	−0.182 **	0.269 **	−0.246 **
	*p*-value	<0.001		<0.001	<0.001	<0.001	<0.001	<0.001
LDL-C	correlation	0.239 **	0.214 **		1.000 **	−0.144 **	0.143 **	−0.165 **
	*p*-value	<0.001	<0.001		<0.001	0.006	0.007	0.002
Trigl	correlation	0.239 **	0.214 **	1.000 **		−0.144 **	0.143 **	−0.165 **
	*p*-value	<0.001	<0.001	<0.001		0.006	0.007	0.002
TSH	correlation	−0.133 *	−0.182 **	−0.144 **	−0.144 **		−0.674 **	0.316 **
	*p*-value	0.011	<0.001	0.006	0.006		<0.001	<0.001
NO	correlation	0.207 **	0.269 **	0.143 **	0.143 **	−0.674 **		−0.445 **
	*p*-value	<.0.001	<0.001	0.007	0.007	<0.001		<0.001

Trigl, triglycerides; oxLDL, oxidised low-density lipoprotein cholesterol; TSH, thyroid-stimulating hormone; UIC, urinary iodine concentration; LDL, low-density lipoprotein; HDL, high-density lipoprotein; NO, nitric oxide. * and ** denote statistical significance.

**Table 5:** Binary logistic regression, univariable and multivariable, Beta coefficients of the factors associated with HDL cholesterol below 40.3 mg/dL.

Variable	Univariable Beta Coefficient (95% CI)	*p* Value	Multivariable Beta Coefficient (95% CI)	*p* Value
Preeclampsia	1.131 (0.973–1.309)	0.102	194.66 (55.15–687.09)	<0.001
TSH > 3 UI/L	1.347 (1.191–1.524)	<0.001	3.98 (1.89–8.40)	<0.001
UIC > 150 µg/L	0.644 (0.566–0.733)	<0.001	0.067 (0.032–0.140)	<0.001
BMI > 25 Kg/m^2^	1.403 (1.212–1.610)	<0.001	1.24 (0.58–2.63)	0.580

TSH: Thyroid-stimulating hormone; UIC: urinary iodine concentration; BMI: body mass index.

**Table 6:** Rotated component matrix and Eigenvalues for participants with preeclampsia.

Variables in the Rotated Matrix	Components ‡		
	1	2	
UIC	0.686		
Triglycerides	−0.734		
HDL	0.832		
LDL	−0.628		
Oxidized LDL		0.688	
Nitric Oxide		−0.863	
TSH		0.766	
**Component**	**Eigenvalues**	**% variance**	**Total variance**
1	2.103	30.05	30.04
2	1.809	25.84	55.89
All five components with Eigenvalues < 1	2.864	44.11	100.00

‡ Factor loadings with values ≥0.500 are shown only for each component with Eigenvalues > 1.

## Data Availability

The raw data supporting the conclusions of this article are available on the website of the journal.

## Abbreviations

The following abbreviations are used in this manuscript:

BMI	Body Mass Index
HDL-C	High-density lipoprotein cholesterol
LDL-C	Low-density lipoprotein cholesterol
NO	Nitric Oxide
oxLDL	Oxidized low-density lipoprotein
ONOO-	Peroxynitrite
PlGF	Placental Growth Factor
sFlt-1	Soluble fms-like tyrosine kinase-1
sdLDL-C	Small-dense low-density lipoprotein cholesterol
T3	Triiodothyronine
T4	Thyroxine
Trigli	Triglycerides
TSH	Thyroid-stimulating hormone
UIC	Urinary Iodine Concentration
VEGF	Vascular Endothelial Growth Factor
VLDL-C	Very low-density lipoprotein cholesterol
